# Beyond TFRC: The Pivotal Role of mGluR2 in Feline Calicivirus Entry and Replication

**DOI:** 10.3390/vetsci12100980

**Published:** 2025-10-13

**Authors:** Ruibin Qi, Hongtao Kang, Yupeng Yang, Kexin Feng, Zhe Liu, Silu Gao, Qian Jiang, Liandong Qu, Jiasen Liu

**Affiliations:** State Key Laboratory for Animal Disease Control and Prevention, Harbin Veterinary Research Institute, Chinese Academy of Agricultural Sciences, Harbin 150069, China; 82101211251@caas.cn (R.Q.); kanghongtao@caas.cn (H.K.); 82101201225@caas.cn (Y.Y.); 82101212402@caas.cn (K.F.); 82101215629@caas.cn (Z.L.); gaosilu2000@163.com (S.G.); jiangqian@caas.cn (Q.J.)

**Keywords:** feline calicivirus, metabotropic glutamate receptor 2, transferrin receptor, viral internalization

## Abstract

**Simple Summary:**

Feline calicivirus (FCV) is a prevalent virus that induces respiratory and oral diseases in felines and serves as a valuable model for investigating other challenging caliciviruses, such as human norovirus. In previous research, we identified a cellular protein known as transferrin receptor (TFRC) that facilitates the entry of FCV into feline cells; however, we sought to determine whether additional proteins are implicated in this process. To explore this, we examined the role of another protein, metabotropic glutamate receptor 2 (mGluR2), in the context of FCV infection through laboratory techniques designed to assess protein interactions and cellular infection. Our results demonstrated that mGluR2 directly interacts with FCV and enhances the virus’s entry into host cells. Notably, when mGluR2 levels were diminished, FCV exhibited significantly reduced infectivity, even in the presence of TFRC, indicating that mGluR2 operates independently of TFRC. These findings contribute to a deeper understanding of the mechanisms underlying FCV infection in cats and position mGluR2 as a novel target for the prevention of FCV-related diseases. Furthermore, they offer valuable insights for the study of human norovirus, which poses significant research challenges.

**Abstract:**

Feline calicivirus (FCV) is among the few members of the Caliciviridae family that can replicate efficiently in vitro. Our recent studies have found the Transferrin Receptor Protein (TFRC) is an entry receptor that facilitates the internalization of FCV. To explore the potential involvement of additional host factors in conjunction with TFRC during the viral entry process, we identified metabotropic glutamate receptor 2 (mGluR2) as a specific interacting partner for both TFRC and the FCV VP1 protein by Co-IP analysis. Our findings indicate that the downregulation of mGluR2, along with its downstream signaling molecule, Calcium-activated potassium channel subunit alpha-1 (KCa1.1), significantly inhibits FCV replication by impairing viral internalization. Importantly, the knockout of TFRC did not diminish the effects of mGluR2 and KCa1.1 on FCV infection. Furthermore, mGluR2 was found to interact directly with FCV VP1, rather than with TFRC, and the rate of F-actin polymerization induced by FCV infection was reduced solely by the downregulation of mGluR2 protein expression, not by TFRC knockout. These results suggest that mGluR2 may independently mediate FCV internalization, operating independently of TFRC, and plays a critical role in the formation of endocytic vesicles. Overall, the results indicate that multiple host factors, including TFRC and mGluR2, are involved in the internalization of FCV into host cells. Further research is necessary to explore the propagation of other caliciviruses, such as norovirus, in vitro.

## 1. Introduction

Receptors are the key for viruses to enter into the host cells and the studies of the interaction between viruses and receptors not only help to understand the pathogenic mechanism of viruses but also facilitate the prevention of viral infections in hosts [1,2]. The Caliciviridae family comprises important pathogens with a broad host range and most members of Caliciviridae have no suitable platform for in vitro culture which greatly restrict the studies of viral receptors participating in calicivirus infection [3,4,5]. Numerous studies have demonstrated the diversity of viral receptors among different genera within the Caliciviridae family. For instance, Caliciviruses can interact with various carbohydrates, including histo-blood group antigens (HBGAs), sialic acid, and heparan sulfate proteoglycans, as well as proteins such as CD300lf and JAM-A, to facilitate cellular binding [6,7,8,9]. The clathrin-mediated endocytosis (CME) pathway is the primary mechanism by which Caliciviruses enter host cells following attachment [10]. However, the precise mechanisms by which Caliciviruses regulate the CME pathway remain to be elucidated.

As one of the very few caliciviruses that can efficiently replicate in vitro, Feline calicivirus (FCV) offers unique advantages for basic research on the Caliciviridae family. The exploration of FCV receptors could help control the FCV infection and successful culture of other caliciviruses in vitro. Junctional adhesion molecule A (JAM-A) serves as the functional receptor for FCV and facilitates the attachment of the virion to host cells [11,12]. And the knockout of JAM-A only has impact on the attachment of FCV to cells which indicates other host factors expert JAM-A mediating the internalization of FCV. Our previous research demonstrated that TFRC can directly interact with FCV VP1 and plays a role in the internalization stage of FCV. However, knocking out TFRC only inhibited, but did not completely block, the entry of FCV into F81 cells. This led us to hypothesize that FCV utilizes multiple host factors as internalization receptors. Furthermore, it remains to be determined whether other host factors are involved in TFRC-mediated FCV internalization.

Metabotropic glutamate receptor 2 (mGluR2), a member of the G protein-coupled receptor (GPCR) family, plays a pivotal role in the regulation of glutamatergic neural signaling. Previous studies have elucidated its involvement in viral entry by modulating membrane fluidity during the endocytosis of influenza viruses, coronaviruses, and other viral entities, facilitated by TFRC [13,14]. As a crucial downstream signaling molecule of mGluR2, the calcium-activated potassium channel subunit alpha-1 (KCa1.1) is capable of modulating intracellular calcium ion concentrations to regulate the activity of endocytosis-related proteins, such as F-actin, thereby mediating the internalization stage of viral entry [14].

In this study, we demonstrated that mGluR2 can also mediate the internalization of FCV in F81 cells by influencing the activity of KCa1.1, in addition to TFRC. Moreover, mGluR2 can cooperate with KCa1.1 to facilitate FCV entry into host cells independently of TFRC. Additionally, mGluR2 was found to specifically interact with both VP1 and TFRC. Collectively, these findings provide further insights into the interactions between FCV virions and viral receptors, including mGluR2 and TFRC. These findings not only enhance our understanding of the molecular mechanism underlying “attachment-endocytosis” in FCV by elucidating the ability of caliciviruses to adapt to host cells through “multi-receptor coordination,” thereby offering a framework for comprehending receptor selection in other challenging-to-culture caliciviruses such as noroviruses; but also identify novel targets for the prevention and control of FCV.

## 2. Material and Methods

### 2.1. Cell Culture and Virus

The F81, F81-c-Flag-TFRC, F81-TFRC^−/−^ and HEK293T cell lines were maintained at 37 °C in a 5% CO2 atmosphere using Dulbecco’s Modified Eagle Medium (DMEM; Gibco, Grand Island, NY, USA, Catalog No. C11995500CP) supplemented with 10% fetal bovine serum and 1% penicillin (100 U/mL) and streptomycin (100 μg/mL). The F81 cell line (RRID: CVCL_9259) was obtained in 2023 from the Kunming Cell Bank of the Chinese Academy of Sciences (KCB) and has been confirmed to be free from mycoplasma contamination, as well as authenticated through species identification. The FCV 2280 strain was preserved at the Harbin Veterinary Research Institute.

### 2.2. Reagents and Antibodies

The study utilized various antibodies and reagents, including the monoclonal anti-Flag^®^ M2 antibody produced in mouse (Sigma-Aldrich, St. Louis, MO, USA, Catalog No. F1804), HA tag polyclonal antibody produced in rabbit (Proteintech Group, Inc., Rosemont, IL, USA, Catalog No. 51064-2-AP), rabbit monoclonal antibody against mGluR2 (Thermo Fisher, Waltham, MA, USA, Catalog No. 702952), and KCa1.1 polyclonal antibody produced in rabbit (Abmart, Shanghai, China, Catalog No. TD8570). Additionally, the study employed anti-mouse IgG (H+L) antibody, Human Serum Adsorbed, DyLight^TM^ 800-Labeled (SeraCare, Milford, MA, USA, Catalog No. 5230-0415), and anti-rabbit IgG (H+L) antibody, DyLight^TM^ 800-Labeled (SeraCare, Milford, MA, USA, Catalog No. 5230-0412).

### 2.3. Plasmid Constructs

The pCMV-HA-C and pcaggs-C-Flag vectors were utilized to construct mammalian expression systems, while the pGEX-4T-1 vector was employed to facilitate protein expression in competent E. coli BL21-CodonPlus (DE3) cells. The gene sequences for feline mGluR2 (XM_006928832.5) and KCa1.1 (XM_011287302.4) were amplified using reverse transcription polymerase chain reaction (RT-PCR) from an F81 cell DNA library.

### 2.4. The Potential TFRC-Binding Proteins Identified by Co-IP Followed by LC-MS Assays

After a 24 h culture period in 10 cm^2^ dishes, F81 and F81-c-Flag-TFRC cells were infected with the FCV 2280 strain at a multiplicity of infection (MOI) of 10. Three hours post-infection, host proteins from both FCV-infected and F81-c-Flag-TFRC cells were extracted using 500 μL of NP-40 lysis buffer to conduct a Co-immunoprecipitation (Co-IP) assay with 20 μL of Anti-Flag^®^ M2 Magnetic Beads (Sigma-Aldrich, St. Louis, MO, USA, Catalog No. M8823). The Co-IP complexes were washed with 500 μL of NP40 with three times and analyzed with Sodium Dodecyl Sulfate-Polyacrylamide Gel Electrophoresis (SDS-PAGE) to identify the distinct protein bands present in F81 cells infected with FCV 2280 compared to F81-c-Flag-TFRC cells infected with FCV or F81-c-Flag-TFRC cells infected with PBS. Subsequently, the unique protein bands were characterized through Liquid Chromatograph Mass Spectrometer (LC-MS) analyses.

For the detection of proteins in gels utilizing LC-MS, proteins are initially digested with trypsin. The resulting peptides are then separated using an Easy nLC system (Thermo Fisher Scientific, Waltham, MA, USA, Catalog No. 1200), which is equipped with a Thermo Acclaim PepMap RSLC column. The separation employs mobile phases consisting of 0.1% formic acid in water and 0.1% formic acid in 80% acetonitrile, with elution occurring at a flow rate of 300 nL/min according to a specified gradient. Detection is performed using a Q Exactive HF-X (Thermo Fisher Scientific, Waltham, MA, USA) mass spectrometer. Subsequent protein identification is achieved through data processing with Proteome Discoverer 2.2 software, in conjunction with a MASCOT 2.6 database search, ensuring a false discovery rate (FDR) of less than 0.01.

### 2.5. Small-Interfering RNA (siRNA) Transfection

A 30 pmol quantity of Small interfering RNAs (siRNAs) targeting mGluR2 and KCa1.1, along with scrambled siRNA controls, was obtained from GenePharma Co., Ltd. (Suzhou, China) and subsequently transfected into cells using 6 μL of the siRNA-mate plus reagent, in accordance with the manufacturer’s protocol.

### 2.6. Quantitative Reverse Transcription PCR (qRT-PCR)

Total RNA was isolated from the cells utilizing the AxyPrep Multisource Total RNA Kit (AxyPrep, Union City, CA, USA, Catalog No. AP-MN-MS-RNA) and was reverse transcribed into complementary DNA (cDNA) using the PrimeScript^TM^ RT Reagent Kit with gDNA Eraser (Perfect Real Time) (Takara, Otsu, Shiga, Japan, Catalog No. RR047A). Quantitative real-time PCR was performed on a QuantStudio 5 Fluorescent Quantitative PCR Instrument (Applied Biosystems, Waltham, MA, USA), employing the ChamQ Universal SYBR qPCR Master Mix (Vazyme Biotech Co., Nanjing, Jiangsu, China, Catalog No. Q711). The primer sequences used in the analysis are provided in Table S1.

### 2.7. Western Blotting (WB)

After treatment, the cells were lysed in each well using 200 μL of RIPA lysis buffer (Beyotime Biotechnology, Haimen, Jiangsu, China, Catalog No. P0013B). The total cell lysates were then subjected to boiling following the addition of loading buffer to prepare for SDS-PAGE electrophoresis. Primary antibodies, diluted according to the specified protocols, were incubated for 16 h. Subsequently, secondary antibodies (Goat anti-Rabbit or Mouse IgG (H+L) Highly Cross-Adsorbed Secondary Antibody), diluted at a ratio of 1:10,000, were incubated for 1 h. Image visualization was performed using an Infrared Imaging System (LI-COR, Lincoln, NE, USA, Catalog No. Odyssey CLX).

### 2.8. Viral Infection

After a 48 h culture period, the cells were infected with the FCV 2280 strain at various multiplicities of infection (MOIs). The entire cell population was harvested 12 h post-infection to evaluate VP1 expression through qRT-PCR and WB assays.

### 2.9. Measurement of Attachment and Internalization Rates

For virus attachment studies, the cells were infected with FCV 2280 at an MOI of 10 and incubated at 4 °C for 1 h. Following synchronized adsorption for 1 h, unbound FCV virions were removed by washing with Phosphate-Buffered Saline (PBS), and the relative levels of bound FCV virions were quantified using qRT-PCR. To assess internalization rates, cells with bound FCV were incubated at 37 °C, washed with acidic PBS (pH 2.5) to eliminate any remaining attached FCV virions, and subsequently analyzed by qRT-PCR.

### 2.10. Co-Immunoprecipitation (Co-IP) Analysis

Following a 24 h incubation period in a 6-well plate, F81 cells were co-transfected with 2 μg of HA/Flag-tagged eukaryotic expression vectors or blank control vectors. Forty-eight hours post-transfection, the F81 cells were lysed using 200 μL of NP-40 to extract total proteins for subsequent co-immunoprecipitation (Co-IP) analysis with 20 μL of Anti-Flag^®^ M2 Magnetic Beads (Sigma-Aldrich, St. Louis, MO, USA, Catalog No. M8823). The Co-IP complexes underwent three washes with 500 μL of NP-40 prior to Western blot (WB) analysis.

### 2.11. Glutathion-S-Transferase (GST) Pull-Down Assay

Recombinant proteins Gst-2280 VP1, Gst-TFRC, and Gst were expressed and purified using BeyoMag^TM^ GSH Magnetic Agarose Beads (Beyotime, Haimen, Jiangsu, China, Catalog No. P2258). After purification and dialysis, 25 μg of each recombinant protein was incubated with 25 μL of BeyoMag™ GSH Magnetic Agarose Beads to perform Gst pull-down assays. Subsequently, total cellular proteins or Flag-tagged mGluR2 and KCa1.1 proteins were extracted from F81 cells and incubated with the GSH beads conjugated to the recombinant proteins. Following an overnight incubation at 4 °C, the pull-down complexes were washed with NP-40 and analyzed via WB analysis.

### 2.12. F-Actin Polymerization Assay

F-actin, or filamentous actin, constitutes a fundamental element of the cytoskeleton. It is generated through the dynamic polymerization of G-actin, which are globular actin monomers, and exhibits the reversible characteristic of “assembly–disassembly”. Through its dynamic structural modifications, F-actin facilitates the spatiotemporal precision and efficiency of the endocytosis process [15,16]. To detect the F-actin polymerization rates, the cells were treated and infected with FCV 2280 at an MOI of 10 and incubated at 37 °C for 30 min to assess F-actin polymerization rates. At 30 min post-infection, the cells were fixed with 4% paraformaldehyde and subsequently washed three times with PBS. The cells were then incubated with phalloidin iFluor^TM^ 488 for 1 h. The viral internalization assay was examined utilizing a Confocal Laser Scanning Microscope (ZEISS, Oberkochen, Baden-Württemberg, Germany, Catalog No. LSM980) equipped with Fast Airyscan technology to assess F-actin polymerization rates. Post-acquisition of confocal images, the fluorescence intensity of F-actin was quantified using Image J software (version 1.8.0, National Institutes of Health, Bethesda, MD, USA) in accordance with the standard intensity analysis protocol. Regions of interest (ROIs) were meticulously selected to minimize background interference, and the mean fluorescence intensity within each ROI was calculated for subsequent statistical analysis.

### 2.13. Statistics

Statistical analyses were conducted using the student’s *t*-test and one-way analysis of variance (ANOVA) with GraphPad Prism software (version 10). Statistical significance was defined as *p*-values less than 0.05, indicating significance, and *p*-values less than 0.01, indicating high significance.

## 3. Results

### 3.1. Identification of Host Proteins Interacting with TFRC

**Figure 1:**
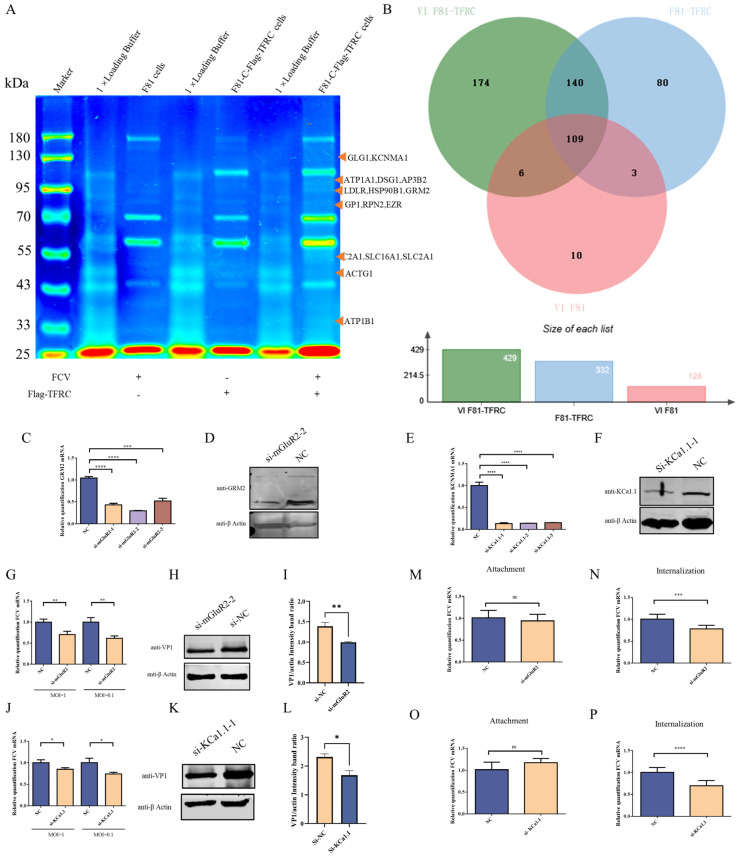
LC-MS analysis of host proteins interacting with TFRC and the effect of those factors on FCV infection. (**A**) The SDS-PAGE analysis demonstrates the interaction of host proteins with TFRC in the presence and absence of FCV infection. (**B**) The Venn diagram illustrates the shared and unique interacting proteins. (**C**) The siRNA-mediated downregulation of mGluR2 mRNA in F81 cells was conducted. (**D**) The siRNA-mediated downregulation of mGluR2 protein in F81 cells was assessed (Original image is in Supplementary Materials Figure S1). (**E**) The siRNA-mediated downregulation of KCa1.1 mRNA in F81 cells was performed. (**F**) The siRNA-mediated downregulation of KCa1.1 protein in F81 cells was evaluated (Original image is in Supplementary Materials Figure S2). (**G**) The number of FCV viral genome copies (VP1) was reduced in F81 cells transfected with siRNA targeting mGluR2. (**H**) The expression of FCV VP1 protein was diminished in F81 cells transfected with siRNA targeting mGluR2 (Original image is in Supplementary Materials Figure S3). (**I**) The gray values from Figure (**H**) were quantitatively analyzed using ImageJ software (version 1.8.0). (**J**) The quantity of FCV viral genome copies (VP1) was decreased in F81 cells transfected with siRNA targeting KCa1.1. (**K**) The expression levels of the FCV VP1 protein were significantly reduced in F81 cells transfected with siRNA targeting KCa1.1 (Original image is in Supplementary Materials Figure S4). (**L**) The gray values obtained from Figure (**K**) were analyzed and compared using ImageJ software. (**M**,**N**) mGluR2 is implicated in the internalization of FCV in F81 cells, rather than in its attachment. (**O**,**P**) KCa1.1 is also involved in the internalization of FCV in F81 cells, rather than its attachment. * *p* < 0.05, ** *p* < 0.01, *** *p* < 0.001, **** *p* < 0.0001, ns: not significant (*p* ≥ 0.05). All statistical significances were determined by the student’s *t*-test and one-way ANOVA unless otherwise stated.

Our prior investigations have demonstrated that TFRC is integral to the internalization phase of the FCV life cycle through its direct interaction with the FCV structural protein VP1. Nonetheless, the involvement of additional proteins in this process remains to be elucidated. To investigate this, a Co-IP assay was conducted to identify potential host proteins interacting with TFRC in F81 cells, under both FCV-infected and uninfected conditions. The results indicated that 140 host proteins commonly interact with TFRC, while 174 host proteins uniquely associate with TFRC during FCV infection (Figure 1A,B) (Original dates were in Supplementary Materials Table S2). Notably, among these proteins, mGluR2, HSPA5, LDLR, and ITGB2 have been implicated in facilitating the entry stage of various viruses, such as SARS-CoV-2, Porcine Epidemic Diarrhea Virus (PEDV), and Crimean-Congo Hemorrhagic Fever Virus (CCHFV).

### 3.2. mGluR2 and KCa1.1 Modulate the Replication of FCV by Regulating Its Internalization into Host Cells

To assess the influence of mGluR2 on FCV replication, three distinct siRNAs specifically targeting mGluR2 were designed and synthesized to suppress mGluR2 protein expression in F81 cells. The results demonstrated that the siRNAs effectively down-regulated both mRNA and protein expression levels of mGluR2 (Figure 1C,D). Consequently, the levels of VP1 protein and viral cDNA copies were significantly diminished following the reduction in intracellular mGluR2 protein expression (refer to Figure 1G–I). To further elucidate the role of mGluR2 in FCV entry, additional analyses were performed. The findings indicated that mGluR2 facilitates the internalization of the virus into cells without influencing viral attachment (Figure 1M,N).

During influenza virus infection, mGluR2 could modulate the activity of KCa1.1, thereby influencing the internalization stage of the virus by affecting F-actin polymerization rates. To explore whether a comparable mechanism is involved in the entry of FCV into host cells, the mRNA and protein expression levels of KCa1.1 in F81 cells were effectively downregulated using siRNA targeting KCa1.1 (Figure 1E,F). This downregulation of KCa1.1 expression subsequently inhibited FCV replication, as evidenced by qRT-PCR and WB analyses (Figure 1J–L). Similar to the role of mGluR2, KCa1.1 appears to influence the internalization rather than the attachment phase of FCV infection (Figure 1O,P). Collectively, these findings indicate that mGluR2 may collaborate with TFRC to facilitate the internalization of FCV by modulating KCa1.1 activity.

### 3.3. The Interaction Analysis Among mGluR2, KCa1.1, TFRC and FCV VP1

**Figure 2:**
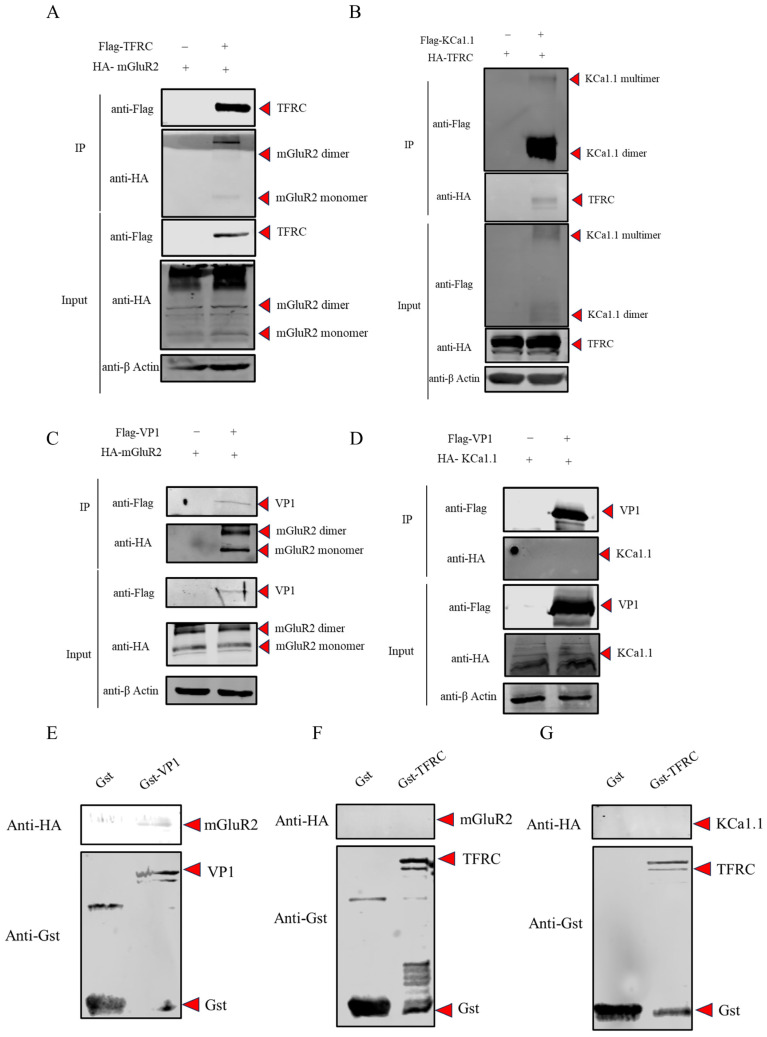
The interaction analysis among mGluR2, KCa1.1, TFRC and FCV VP1. (**A**) Co-IP assays demonstrated that mGluR2 interacts with TFRC. (**B**) KCa1.1 was also shown to interact with TFRC through Co-IP assays. (**C**) Additionally, FCV VP1 was found to interact with mGluR2 as determined by Co-IP assays. (**D**) In contrast, no interaction was observed between FCV VP1 and KCa1.1 in Co-IP assays. (**E**) A direct interaction between FCV VP1 and mGluR2 was confirmed via GST pull-down assays. (**F**) No direct interaction was detected between TFRC and mGluR2 in GST pull-down assays. (**G**) Similarly, no direct interaction was observed between TFRC and KCa1.1 in GST pull-down assays (Original image is in Supplementary Materials Figures S5–S11).

To gain a more comprehensive understanding of the molecular mechanisms underlying the interaction between mGluR2 and the FCV entry stage, we employed Co-IP assays to investigate the interactions among mGluR2, FCV VP1, TFRC, and KCa1.1. The results indicated that TFRC interacts with both mGluR2 and KCa1.1 (Figure 2A,B), consistent with the findings from LC-MS assays identifying potential host protein interactions with TFRC. However, only mGluR2 was found to interact with FCV VP1 (Figure 2C,D), suggesting that mGluR2 may function as an entry receptor for FCV.

To further investigate whether mGluR2 could serve as an internalization receptor during FCV infection, GST pull-down assays were conducted to assess the direct interactions between mGluR2 and FCV VP1, as well as between mGluR2 or KCa1.1 and TFRC. The assays revealed that only mGluR2 directly interacts with FCV VP1 (Figure 2E), while TFRC does not directly interact with either mGluR2 or KCa1.1 (Figure 2F,G). These findings suggest that mGluR2 collaborates with KCa1.1 to mediate FCV internalization, a process that does not necessitate the involvement of TFRC.

### 3.4. mGluR2 Mediates FCV Internalization Without TFRC Involved

**Figure 3:**
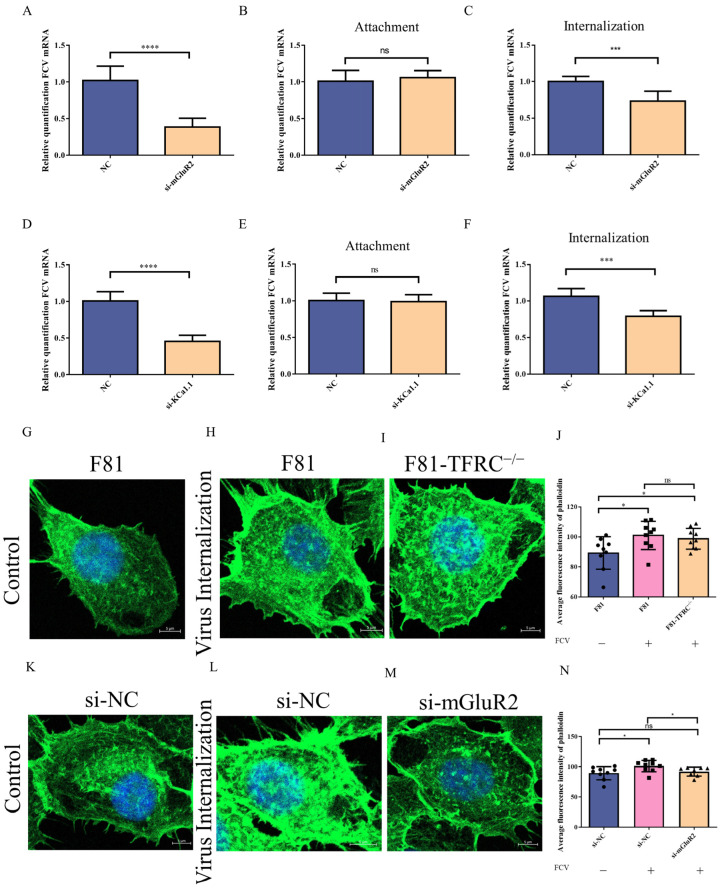
mGluR2 mediates FCV facilitates the internalization of FCV independently of TFRC involvement. (**A**) The absence of TFRC does not impede the influence of mGluR2 on FCV replication. (**B**,**C**) Similarly, the absence of TFRC does not hinder the effect of mGluR2 on FCV entry. (**D**) The absence of TFRC does not affect the impact of KCa1.1 on FCV replication. (**E**,**F**) Consistent with previous results, the absence of TFRC does not impede the effect of mGluR2 on FCV entry. (**G**–**J**) The knock-out of TFRC does not influence the polymerization rates of F-actin. (**K**–**N**) The down-regulation of mGluR2 inhibits the polymerization rates of F-actin. * *p* < 0.05, *** *p* < 0.001, **** *p* < 0.0001, ns: not significant (*p* ≥ 0.05). All statistical signifi-cances were determined by the student’s *t*-test and one-way ANOVA unless otherwise stated.

Previous research has demonstrated that TFRC is essential for agonist-induced endocytosis of mGluR2. To investigate whether a similar mechanism is present during FCV infection, siRNA targeting mGluR2 and KCa1.1 was transfected into F81-TFRC^−/−^ cells to assess the role of TFRC in FCV internalization mediated by mGluR2 and KCa1.1. The results indicated that downregulation of mGluR2 or KCa1.1 protein expression inhibited FCV replication or internalization in F81 cells lacking TFRC expression (Figure 3A,C,D,F), consistent with observations in F81 cells. Furthermore, the attachment rates of FCV virions in F81-TFRC^−/−^ cells were not affected by modulation of mGluR2 or KCa1.1 protein expression (Figure 3B,E). Consequently, we propose that mGluR2 and KCa1.1 independently modulate the FCV entry process without reliance on TFRC.

To further validate our conclusions, phalloidin iFluor^TM^ 488 was employed to assess the regulation of F-actin polymerization rates by FCV infection and siRNA interference. The results demonstrated that FCV infection led to an increase in F-actin polymerization rates in F81 cells, regardless of whether they were transfected with control siRNA (Figure 3G,H,K,L). However, knockdown of TFRC did not significantly affect the FCV-induced increase in F-actin polymerization rates (Figure 3I). Furthermore, the reduction in mGluR2 protein expression inhibited the F-actin polymerization induced by FCV infection (Figure 3M). These findings suggest that mGluR2 facilitates FCV internalization by enhancing the activity of KCa1.1, thereby promoting F-actin polymerization.

## 4. Discussion

The clathrin-mediated endocytosis (CME) pathway is a ubiquitous mechanism employed by a wide variety of viruses, including feline calicivirus (FCV), to gain entry into host cells [10,16,17,18]. The formation of clathrin-coated vesicles (CCVs) represents a crucial initial phase of CME, which can occur spontaneously or be induced by viral infections, such as those caused by the influenza virus and reovirus [19,20,21]. The majority of CME processes necessitate the involvement of the Adaptor Protein 2 (AP2) complex, with the YXXΦ motif serving as a pivotal functional domain that facilitates interactions between the AP2 complex and other associated proteins [22,23]. Viruses such as Hepatitis B Virus (HBV) and White Spot Syndrome Virus (WSSV), whose structural proteins contain the YXXΦ motif, can directly engage with the AP2 complex to initiate CME [24,25]. Conversely, viruses lacking the YXXΦ motif, such as FCV, can interact with host membrane receptor proteins to recruit the AP2 complex and trigger CME [26].

In this study, we identified another FCV entry receptor, mGluR2, besides TFRC and verified mGluR2 could specifically interact with FCV VP1 and TFRC. To further exploit the mechanisms of mGluR2 mediating FCV internalization, KCa1.1 was also down-regulated by siRNA, and we found it influences FCV replication by mediating viral internalization stage and could not interact with FCV VP1. This indicated KCa1.1 is likely to be regulated by mGluR2 to effect FCV entry instead of directly interacting with FCV VP1. The mechanism by which KCa1.1 regulate viral entry has been demonstrated in Influenza virus infection [14]. And our studies also showed that FCV can use a similar mechanism, as evidenced by the finding that down-regulation of mGluR2 can inhibit the F-actin polymerization rates induced by FCV infection.

It has already been confirmed that TFRC can cooperate with mGluR2 to mediate the internalization of various enveloped viruses such as Rabies Virus and SARS-CoV-2 [13,27]. Most GPCR family members undergo endocytosis through the CME pathway [28,29]. Additionally, mGluR2 could directly interact with TFRC by Gst pull-down analysis and co-localize with TFRC in early and late endosomes [13]. In our study, we did not observe a direct interaction between feline mGluR2 and TFRC, as evidenced by the detection of only weak band signals in the Co-IP analysis. The presence of a faint mGluR2-TFRC band in the Co-IP assay suggests that the interaction may be indirect, potentially mediated by intermediary proteins. Typically, direct interactions result in the formation of stable complexes that produce strong signals, whereas indirect interactions are characterized by multi-component complexes that are susceptible to dissociation during lysis and washing procedures due to their weaker affinity. This dissociation likely leads to reduced precipitate formation and subsequently weaker signals. The observed phenomenon may indicate a level of regulatory complexity, wherein intermediary proteins modulate the dynamics of mGluR2-TFRC interactions to prevent excessive binding and optimize the efficiency of viral internalization and replication. Furthermore, the knockout of TFRC did not affect the role of mGluR2 in FCV entry, suggesting that mGluR2 modulates FCV entry independently of TFRC, a mechanism that appears to differ from those observed in other viruses.

This study identifies mGluR2 and KCa1.1 as novel therapeutic targets for FCV inhibition, given their specificity for the internalization step—a critical vulnerability in the viral life cycle. Further work is needed to: (1) map the interacting domains of mGluR2 and VP1 to enable the design of blocking peptides; (2) define the signaling cascade linking mGluR2 activation to KCa1.1-mediated F-actin polymerization; and (3) explore whether other caliciviruses (e.g., noroviruses) utilize similar mGluR2-KCa1.1-dependent entry mechanisms, which would highlight a conserved viral strategy.

## 5. Conclusions

In summary, our research elucidates a novel entry pathway for feline calicivirus (FCV) that operates independently of transferrin receptor protein 1 (TFRC) and is mediated by the metabotropic glutamate receptor 2 (mGluR2) and large conductance calcium-activated potassium channel (KCa1.1) axis. This discovery significantly enhances our comprehension of host–virus interactions in caliciviruses and highlights mGluR2 and KCa1.1 as potential therapeutic targets for the prevention and control of FCV. Future investigations will aim to map the interaction domain between mGluR2 and the viral protein VP1, delineate the complete signaling cascade that connects mGluR2 to F-actin polymerization, and examine the conservation of this pathway in other caliciviruses, such as noroviruses.

## Data Availability

The original contributions presented in this study are included in the article/Supplementary Materials. Further inquiries can be directed to the corresponding author.

## Supplementary Materials

The following supporting information can be downloaded at: https://www.mdpi.com/article/10.3390/vetsci12100980/s1, Table S1. The qRT-PCR primer sequences of this manuscript. Table S2. The proteins potentially interacting with TFRC in F81 cells infected with FCV were identified through LC-MS analysis. Figure S1. Original data of siRNA-induced mGluR2 protein downregulation assessment in F81 cells. Figure S2. Original data of siRNA-induced KCa1.1 protein downregulation assessment in F81 cells. Figure S3. Original data for detecting FCV VP1 protein expression reduction in F81 cells transfected with mGluR2-targeting siRNA. Figure S4. Original data for detecting FCV VP1 protein expression reduction in F81 cells transfected with KCa1.1-targeting siRNA. Figure S5. Original data showing mGluR2-TFRC interaction via Co-IP assays. Figure S6. Original data showing KCa1.1-TFRC interaction via Co-IP assays. Figure S7. Original data showing mGluR2- FCV VP1 interaction via Co-IP assays. Figure S8. Original Co-IP assay data showing no interaction between FCV VP1 and KCa1.1. Figure S9. Original data of GST pull-down assays confirming direct interaction between FCV VP1 and mGluR2. Figure S10. Original data of GST pull-down assays detecting no direct interaction between TFRC and mGluR2. Figure S11. Original data of GST pull-down assays showing no direct interaction between TFRC and KCa1.1.
