# Supplementary material for: Beyond TFRC: The Pivotal Role of mGluR2 in Feline Calicivirus Entry and Replication
Source: Vet Sci. 2025 Oct 13;12(10):980. doi: 10.3390/vetsci12100980 (PMC12567643; doi:10.3390/vetsci12100980)
Supplement: Supplementary file 1 [file vetsci-12-00980-s001.zip › Table S1.pdf]

Primer Name	Sequence (5’ to 3’)	Size
qPCR-GAPDH-F	AATTCCACGGCACAGTCAAGGC	124 bp
qPCR-GAPDH-R	ACAACATACTCAGCACCAGCATCAC	
qPCR-FCV-F	TCAAACTCTGAGCTTCGTGC	174 bp
qPCR-FCV-R	CAGTCAGGACAAACGTCATAAC	
qPCR-mGluR2-F	TCCTGCTGGGCGGTGTCTTC	150 bp
qPCR-mGluR2-R	CGATGCGGTTGGTCTTGGTGAG	
qPCR-KCa1.1-F	GCGGAGGCAGCAGTCTTAGAATG	87 bp
qPCR-KCa1.1-R	AGGAGGAAGAGGAGGAGGAGGAG	
