# Supplementary figures and images for: Beyond TFRC: The Pivotal Role of mGluR2 in Feline Calicivirus Entry and Replication
Source: Vet Sci. 2025 Oct 13;12(10):980. doi: 10.3390/vetsci12100980 (PMC12567643; doi:10.3390/vetsci12100980)

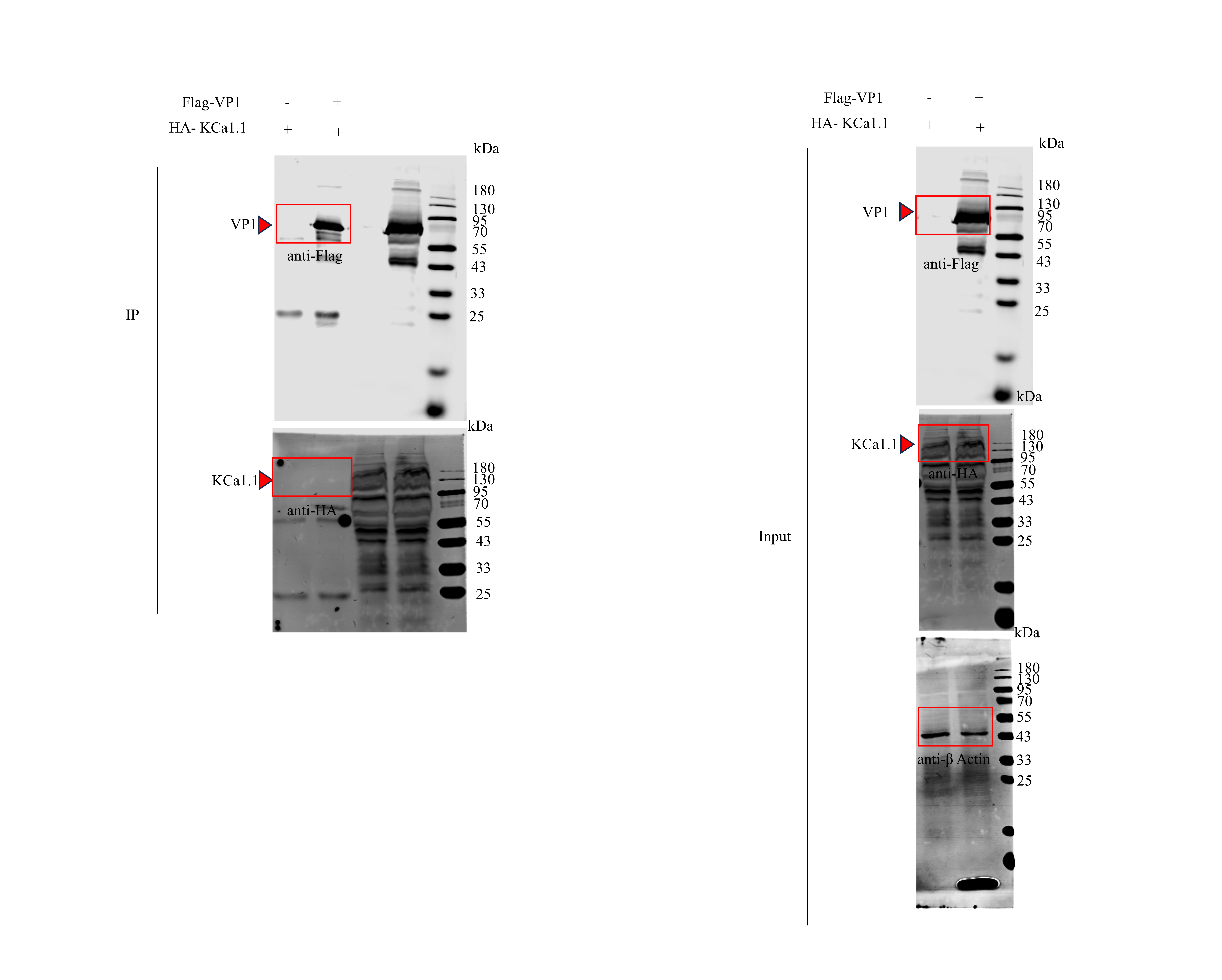

Supplement: Supplementary file 1 [file vetsci-12-00980-s001.zip › Original Images for Blots/Figure S8.tif]
